# Effects of dinotefuran and its metabolites on the early life stage development of medaka (*Oryzias latipes*): *In vivo* and *in silico* studies

**DOI:** 10.1016/j.toxrep.2026.102293

**Published:** 2026-06-12

**Authors:** Masashi Hirano, Daishi Inoue, Masaya Uchida, Keisuke Takahashi, Keisuke Kato, Tadashi Okobira, Hiroshi Ishibashi, Koji Arizono, Nobuaki Tominaga

**Affiliations:** a Department of Food and Life Sciences, School of Agriculture, Tokai University, 871-12 Sugido, Mashiki-Machi, Kamimashiki-Gun, Kumamoto 861-2205, Japan; b Department of Creative Engineering, National Institute of Technology, Ariake College, 150 Higashi-Hagio, Omuta, Fukuoka 836-8585, Japan; c Faculty of Pharmaceutical Sciences, Toho University, 2–2-1 Miyama, Funabashi, Chiba 274–8510, Japan; d Graduate School of Agriculture, Ehime University, 3-5-7 Tarumi, Matsuyama, Ehime 790-8566, Japan; e Graduate School of Pharmaceutical Sciences, Kumamoto University, 5–1 Oe, Chuo-ku, Kumamoto, Kumamoto 862-0973, Japan

**Keywords:** Neonicotinoid insecticides, Dinotefuran and its metabolites, Developmental toxicity, *Oryzias latipes*, Molecular docking

## Abstract

Dinotefuran (DINO), a widely used neonicotinoid insecticide, has attracted significant attention because of its environmental risks. Although DINO is metabolized in the environment, its specific toxicological risks remain unclear. In this study, we aimed to determine the effects of DINO and its metabolites, 2-nitro-1-(tetrahydro-3-furylmethyl)guanidine (FNG), 1-methyl-3-(tetrahydro-3-furylmethyl) guanidine (DN), 1-methyl-3-(tetrahydro-3-furylmethyl)-urea (UF), and N-aminodinotefuran (NAD) on the embryonic development of medaka (*Oryzias latipes*), as incorporated by a nanosecond pulsed electric field (nsPEF) technique. No acute toxicity leading to death was observed during the early developmental stages of medaka exposed to DINO and its metabolites at concentrations of 1 and 2 mM; however, some larvae malformed after hatching, indicating developmental effects caused by DINO and its metabolites. Approximately 10% of the larvae in all the treatment groups were malformed after hatching, with significant differences observed in the 2 mM NAD treatment group. Transcriptome sequencing revealed that the neuroactive ligand–receptor interaction pathway plays an important role in DINO-induced developmental toxicity. Furthermore, we constructed *in silico* homology model of the medaka α4β2 nicotinic acetylcholine receptor (nAChR). Molecular docking simulation revealed that DINO metabolites bind stably to nAChR. The order of the interaction potentials of DINO with medaka α4β2 nAChR was as follows: NAD > DINO > DN > FNG > UF. Overall, our findings provide fundamental data regarding the biological roles of DINO and its metabolites in the developmental toxicity responses of fish to neonicotinoids.

## Highlights

• Dinotefuran and its metabolites are not acutely toxic to medaka embryos.
• Dinotefuran and its metabolites adversely affect larval development.
• Transcriptomic study identifies gene pathways linked to developmental toxicity.
• Dinotefuran and its metabolites interact with medaka nAChRs.

## Graphical Abstract

**Figure:**
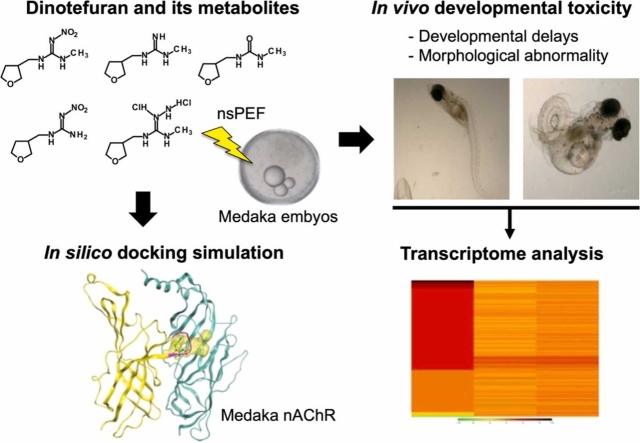

## 1 Introduction

Neonicotinoids are one of the most widely used insecticides worldwide [1], [2], [3]. Neonicotinoids currently account for around 27% of the global insecticide market and are registered for use on over 140 different crops in more than 120 countries. Jeschke et al., [2], [3], [4], [5]. Despite acting as agonists of insect nicotinic acetylcholine receptors (nAChRs), they exhibit low acute toxicity toward vertebrates, thereby posing a low risk to humans and other vertebrates [6]. Their physicochemical properties make them useful in agriculture, as many neonicotinoids are relatively persistent and have high water solubility. Neonicotinoids are absorbed by the roots and translocated to almost all plant tissues, including leaves, flowers, pollen, nectar, and crops, and are commonly used to treat plant seeds [4], [7], [8], [9].

Neonicotinoids are ubiquitous in food, drinking water, and the environment [10], [11]. In the USA, quantitative analysis of food commodities has shown that 72% of fruits and 45% of vegetables contain detectable neonicotinoids, including acetamiprid, dinotefuran (DINO), flonicamid, imidacloprid, thiacloprid, and thiamethoxam [8]. Two recent cross-sectional studies (the US Congressional Cafeteria Study and the Hangzhou China Study) have also reported common food contamination with neonicotinoids [12]. Ikenaka et al. [13] identified seven neonicotinoids (acetamiprid, clothianidin, DINO, imidacloprid, nitenpyram, thiacloprid, and thiamethoxam) commonly shipped to Japan, as well as 10 metabolites found in Japanese tea leaves and beverages. Neonicotinoids accumulate in plant bodies and are transported from agricultural fields to surface waters, thereby increasing public health concerns regarding food contamination and water pollution. Neonicotinoids have been widely detected in the surface waters of the USA, Canada, and Japan [14], [15], [16], [17], [18], [19]. Sultana et al. [20] detected neonicotinoid-derived insecticides in drinking water samples collected from agricultural regions in Canada. Furthermore, neonicotinoids have been detected in urine samples of Japanese children, with DINO showing the highest concentration among the detected neonicotinoids [21]. Thus, these reports indicate that neonicotinoids are prevalent in humans, food products and the environment, including surface waters. This has sparked concerns over their potential impact on ecosystems.

**Fig. 1:**
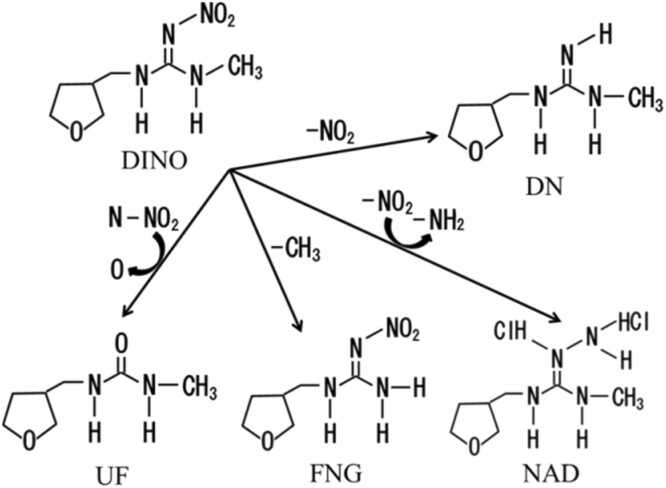
Chemical structures of dinotefuran (DINO) and its metabolites.

DINO, a non-chlorinated neonicotinoid, is shipped to Japan [22]. DINO is among the major neonicotinoid-derived insecticide contaminants in river water and has been detected in over 90% of river sampling points in Saitama and Fukuoka Prefectures in Japan [17], [18]. In addition, concentrations of DINO up to 220 ng/L have been detected in the aquatic environment in Osaka, Japan [23]. Although DINO is relatively stable in the environment (50–100 d half-life), it is primarily degraded via photolysis and microbial activity [24]. The major DINO metabolites are 2-nitro−1-(tetrahydro−3-furylmethyl)guanidine (FNG), 1-methyl−3-(tetrahydro−3-furylmethyl) guanidine (DN), and 1-methyl−3-(tetrahydro−3-furylmethyl)-urea (UF), which are also detected in plants, water, soil, and mammals [25] (Fig. 1). DN is one of the main degradation products, and DN and UF are important metabolites in both plants and animals [26]. These metabolites are highly toxic to soil invertebrates [27]. In Japanese green tea leaves, DINO has been detected at the highest level, and its metabolite, UF, is the most commonly detected among the 20 neonicotinoid metabolites. DINO (59 ng/mL), followed by UF (0.6 ng/mL) and FNG (0.1 ng/mL), exhibits the highest detection frequency and concentration in bottled green tea beverages [13]. Furthermore, Yin et al. [28] indicated that the main metabolites of DINO enantiomers in zebrafish were UF and DN. A recent study found that the average concentration of six neonicotinoid metabolites, including UF, in the effluent of wastewater treatment plants (WWTPs) in Shanghai, China, was 478.2 ng/L. Zhang et al., [29]. Therefore, DINO and its metabolites are among the most abundant neonicotinoids.

A nanosecond pulsed electric field (nsPEF) technique is an electroporation method which utilizes several thousand volts discharged in nanoseconds. It facilitates easy and quantifiable introduction of chemical substances by temporarily altering the permeability of the medaka egg membrane. This technique has improved the study of embryonic developmental toxicity of chemicals in medaka embryos because nsPEF can efficiently deliver substances without damaging the fetal membrane. Our previous studies using nsPEF technique have demonstrated the teratogenicity and embryonic developmental toxicity of metals and chemical substances such as lithium ion, nickel, copper, selenium, 17β-estradiol, equine estrogens and benzo[*a*]pyrene [30], [31], [32], [33], [34], [35]. Thus, nsPEF technology using medaka embryos is efficient in the assessment of teratogenicity and developmental toxicity.

Neonicotinoids exhibit stronger agonist potency for insect nAChRs than vertebrate nAChRs. In contrast, some neonicotinoid metabolites confer differential selectivity to insect and vertebrate nAChRs [6], [36]. Tomizawa et al. [36] indicated that the iminium metabolites of imidacloprid and thiacloprid exhibit a high affinity for vertebrate neuronal α4β2 nAChR. These studies highlight the need to investigate the effects of neonicotinoid metabolites on aquatic vertebrates. To date, only a few studies have evaluated the developmental toxicity of parent compounds and their metabolites in fish [10]. For example, exposure to Rac-dinotefuran, S-dinotefuran and R-dinotefuran altered the development of zebrafish, affecting their body weight and length and resulting in growth delay [37]. Therefore, in the present study, the objective of this study was to investigate the effects of DINO and its metabolites (UF, FNG, and DN) on the embryonic development of medaka (*Oryzias latipes*) using the nsPEF technology. In addition, to investigate the effects of structural differences on activity, we also examined the influence of N-aminodinotefuran (NAD), a related compound. Futhermore, *in silico* docking analysis was performed to better understand the structural characteristics of neonicotinoids binding to medaka nAChR.

## 2 Materials and methods

### 2.1 Test chemicals

DINO (Cas No. 165252–70–0; purity > 99%) was purchased from Wako Pure Chemical Industries, Ltd. (Osaka, Japan). The DINO metabolites (UF, FNG, NAD, and DN; Fig. 1) were synthesized at Toho University in Japan [13].

### 2.2 Animals

Medaka fish were obtained from the National Institute of Environmental Studies (Ibaraki, Japan). The medaka were fed *Artemia nauplii* and a commercial feed (Hikari, Tokyo, Japan) twice daily. The tanks were maintained under a 16/8 h light/dark photoperiod at a temperature of 25 ± 1 °C. The embryos were collected from the breeding tanks, and the fertilized embryos were selected under a digital microscope VHX-900F (Keyence, Osaka, Japan) within 5 h of fertilization [38]. These embryos were then maintained at the same temperature and photoperiod as their parents in preparation for subsequent experiments.

### 2.3 Developmental toxicity assay: chemical incorporation into fertilized medaka eggs using nanosecond pulsed electric field (nsPEF) and morphology observations

Chemical incorporation was performed as previously described [34], [35], with slight modifications. DINO and its metabolites, UF, FNG, DN, and NAD at concentrations of 1 and 2 mM (dissolved in 0.01% dimethyl sulfoxide) were selected based on the effective concentrations in previous studies for various fish species, in order to elucidate their toxicological effects on the embryonic development of medaka [10], [37], [39], [40], [41], [42]. The control group was exposed to 0.01% dimethyl sulfoxide as a solvent control. Fertilized medaka eggs from various breeding pairs were collected within 5 h of fertilization. The fertilized eggs were randomly selected and treated with DINO and its metabolites. Individual medaka eggs were subjected to nsPEF treatment in an isotonic solution in the presence or absence of the indicated concentrations of the chemicals (n = 30/group). The nsPEF treatment was performed by placing one medaka embryo in a cuvette containing 90 μL of exposure solution and applying high electric field pulses at 3.0 kV for 100 nanoseconds at 10-millisecond intervals followed by 30 V for 1 millisecond. After applying nsPEF, the eggs were soaked in the same solution for 2 h and thoroughly washed with an isotonic solution. Six eggs were placed separately in a 5-mL container with the isotonic solution using a six-well plate. During the observation period, the embryos were maintained at 25 ± 1 °C, and the isotonic solution in the wells was changed daily. Developmental abnormalities, mortality, hatching time, and hatchability were recorded daily by using a digital microscope (VHX-900F; Keyence, Osaka, Japan). The lethality of the medaka eggs was calculated 24 h after the nsPEF treatment. Developmental delays and morphological abnormalities including hypercardia (Fig. S1B), thrombosis (Fig. S1C) and deformation (Fig. S1E) are shown in the Supplementary Information.

### 2.4 Measurement of the incorporated DINO concentrations in medaka eggs

A total of 700 medaka eggs were soaked in 2 mM DINO for 2 h, with or without nsPEF treatment. The collected eggs were frozen at −80 °C until analysis. The eggs were then thawed and crushed in ultrapure water using a plastic pestle. Crude medaka egg homogenates were obtained via centrifugation at 14,000 rpm for 10 min at 4 °C and extracted with an equal volume of hexane for delipidation. Aliquots of delipidated homogenates were analyzed using an Acquity UPLC-MS system (Waters Corp., Milford, MA, USA). All the measurement conditions are listed in Table S1.

### 2.5 RNA-sequencing (RNA-seq) analysis

RNA-seq analysis was performed as per the previously reported method [33]. Briefly, medaka embryos were cultured for six days with 2 mM DINO, UF, FNG, DN, and NAD in the treatment groups and 0.01% dimethyl sulfoxide in the solvent control group. This process was repeated several times, and 70 samples were collected. Total RNA was isolated from the medaka embryos using the RNeasy Micro Kit (Qiagen, Hilden, Germany) as previously described [34], [35], [43]. The quantity and the purity of total RNA were examined photometrically by measuring the absorbance at 260:280 and 260:230 nm using a Q5000 spectrophotometer (Tomy Seiko Co., Ltd., Tokyo, Japan), followed by electrophoretic analysis using an RNA 6000 Nano LabChip Kit with an Agilent Bioanalyzer 2100 (Agilent Technologies, Santa Clara, CA, USA). RNA samples with an RNA integrity number (as defined by Agilent Technologies) > 9.2 were used for the RNA-seq analysis. Bioinformatic analyses were conducted using the CLC Genomics Workbench software version 10.1.1 (Qiagen). RNA-seq data were annotated using sequence data from the Ensembl database (https://asia.ensembl.org/index.html). To identify differentially expressed genes, the expression levels of each gene in the control and DINO-treated samples were determined and normalized to reads per kilobase per million. Genes with expression levels more than three times or less than 1/3 that of the nsPEF-treated control were defined as genes with fluctuating expression. Gene Ontology analysis was performed using the Database for Annotation, Visualization, and Integrated Discovery (https://david.ncifcrf.gov/) to analyze the pathways affected by changes in the gene expression.

### 2.6 *In silico* docking studies

Homology modeling of medaka α4β2 nAChR was performed using the molecular operating environment program (version 2019.01, Chemical Computing Group, Inc., Montreal, QB, Canada). Amino acid sequences of medaka nAChR α4 (accession no: XP_023812103.1) and β2 (accession no: XP_023819667.1) were obtained from GenBank (https://www.ncbi.nlm.nih.gov/protein). The crystal structure of the ACh-binding protein of *Lymnaea stagnalis*, in complex with desnitro-imidacloprid (Protein Data Bank ID 3WTN) was downloaded from the Protein Data Bank (http://www.rcsb.org). Homology model of the medaka α4β2 nAChR dimer was generated using chains B and C of 3WTN as the template for dimer modeling, including the ligand. Among the 10,000 intermediate models generated, the structure with the lowest total potential energy was used for the docking studies. The final model was further processed to add hydrogen atoms and to perform energy minimization using the AMBER10:EHT force field [44], [45] and Born solvation.

The two-dimensional structures of DINO, UF, FNG, DN, and NAD were constructed using BIOVIA Draw 2018 (BIOVIA, Dassault Systèmes) and converted to 3D coordinates using Rebuid3D with the AMBER:EHT force field. A chemical library of the 3D structures was constructed. Docking simulations were then performed using the Induced-Fit docking program in a molecular operating environment. Fifty confirmations for each chemical were generated using the triangle matcher method and London DG scoring. For refinement, an induced-fit method with a rigid backbone was used and rescored using the GBVI/WSA dG function. The best positions were determined based on the lowest S-score (kcal/mol).

### 2.7 Statistical analyses

The incidences of developmental abnormalities and malformations were tested for significance using the IBM SPSS statistical analysis software (Tokyo, Japan). Significance was tested using the Kruskal–Wallis multi-group non-parametric test.

Multivariate analysis of RNA-seq data annotated at the National Center for Biotechnology Information was performed via principal component and cluster analyses using CLC Genomics Workbench v10.1.1 (Qiagen), an integrated sequence analysis software.

## 3 Results

### 3.1 DINO concentrations in medaka eggs

To measure the DINO concentrations, medaka eggs were exposed to 2 mM DINO for 2 h with or without nsPEF treatment. In eggs without nsPEF treatment, 137 µM DINO was detected (Fig. S2). In contrast, DINO concentration in the nsPEF-treated eggs was 448 µM (Fig. S2). These results confirm the successful incorporation of DINO into medaka eggs following nsPEF treatment.

### 3.2 Evaluation of the abnormalities, hatching, and deformities in medaka

No significant acute toxicity leading to death was observed in any of the treatment groups. Although few embryos with thrombi were observed in the control and treatment groups, their growth was not affected by DINO. No significant differences were observed in the incidence of thrombosis between the control and treatment groups (Table 1). A few unhatched embryos were observed in both the control and treatment groups. The mean hatching date was approximately 10 d, with no significant differences between the control and treatment groups. These results suggest that DINO metabolites had no effect on egg hatching.

**Table 1:** Average hatching day and thrombus incidence in medaka eggs.

Treatment group (mM)		*n*	Average hatching time (day)	Thrombus (%)
CTL(−)		30	9.52	0.0
CTL(+)		30	9.64	3.3
DINO	1	30	10.00	13.0
	2	30	9.79	6.6
UF	1	30	10.44	10.0
	2	30	9.48	6.6
FNG	1	10	9.97	0.0
	2	10	9.29	3.3
NAD	1	30	10.08	3.3
	2	30	9.59	6.6
DN	2	30	10.24	4.2

CTL: control. (−) = no nsPEF application and (+) = nsPEF application.

**Fig. 2:**
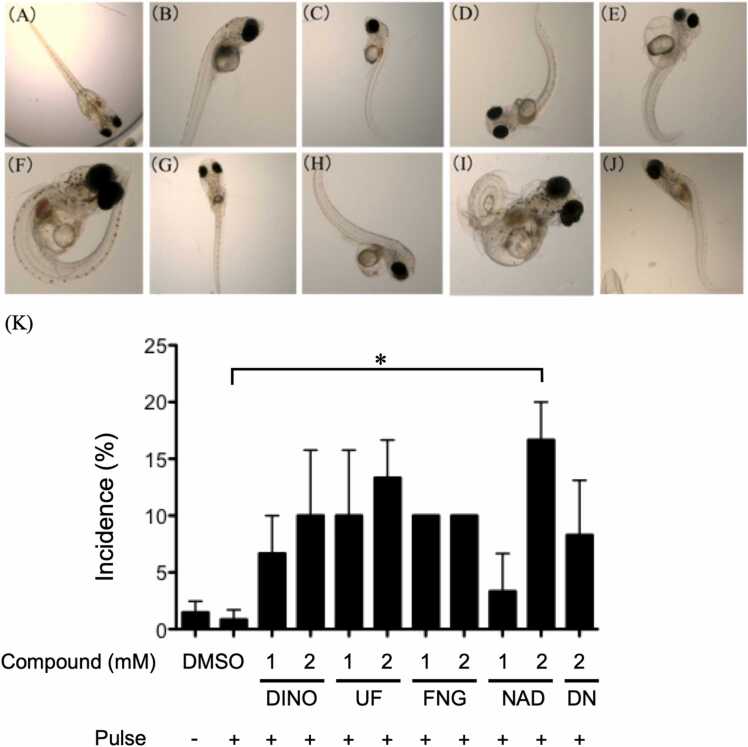
**Malformations in medaka embryos exposed to DINO and its metabolites.** (A) CTL(+). (B) 1 mM DINO. (C) 1 mM 1-methyl−3-(tetrahydro−3-furylmethyl)-urea (UF). (D) 1 mM 2-nitro−1-(tetrahydro−3-furylmethyl)-guanidine (FNG). (E) 1 mM N-aminodinotefuran (NAD). (F) 2 mM DINO. (H) 2 mM FNG. (I) 2 mM NAD. (J) 2 mM 1-methyl−3-(tetrahydro−3-furylmethyl)guanidine (DN). (K) Incidence of medaka larval malformations with each test concentration of DINO and its metabolites (*n* = 30; except *n* = 10 for FNG groups).

The incidence of malformed larvae after hatching was approximately 10% in all the treatment groups, with significant differences between the 2 mM NAD-treated and control groups (Fig. 2). The lateral dorsal (Figs. 2D and 2G), longitudinal (Figs. 2B, 2C, 2H, and 2 J), and caudal (Figs. 2F and 2I) curvatures were the most common symptoms in all treatment groups. Chemical-specific deformities were not observed.

### 3.3 Effects of DINO exposure on medaka embryo transcriptome

RNA-seq analysis was performed to understand the similarities and differences between the transcriptional responses to DINO. We identified 3767 (2773 upregulated and 994 downregulated), 5511 (5004 upregulated and 507 downregulated), 3994 (3273 upregulated and 721 downregulated), 8474 (772 upregulated and 7702 downregulated), and 9065 (8582 upregulated and 483 downregulated) differentially expressed genes in embryos after exposure to DINO, UF, FNG, DN, and NAD, respectively. Functional pathway analysis using the Database for Annotation, Visualization, and Integrated Discovery indicated that neuroactive ligand–receptor interactions were enriched in all the treatment groups. The pathway involved in the cytokine–cytokine receptor interaction was also commonly altered by DINO in all treatment groups, except in the DN group. Steroid hormone biosynthesis, intestinal immune network for IgA production, and retinol metabolism were altered in the DINO and NAD treatment groups (Table 2). In contrast, metabolic pathway, steroid biosynthesis, primary bile acid biosynthesis, pyrimidine metabolism, glutathione metabolism, GPI-anchor biosynthesis, and adipocytokine signaling pathways were altered only in the DN treatment group.

**Table 2:** Pathway analysis of the differentially expressed genes (DEGs) in medaka embryos exposed to dinotefuran (DINO) and its metabolites.

KEGG Entry	Term	DINO				UF				FNG				NAD				DN		
		Count	%	*p*-value		Count	%	*p*-value		Count	%	*p*-value		Count	%	*p*-value		Count	%	*p*-value
ola04080	Neuroactive ligand–receptor interactions	31	7.09	3.66E−09		37	8.89	2.07E−14		50	7.45	3.77E−16		42	8.50	6.35E−14		69	2.96	0.009
ola04060	Cytokine–cytokine receptor interactions	13	2.97	7.48E−05		14	3.37	6.88E−06		14	2.09	8.03E−04		14	2.83	1.24E−04				
ola04514	Cell adhesion molecules (CAMs)									14	2.09	2.41E−02								
ola00140	Steroid hormone biosynthesis	6	1.37	2.34E−03		5	1.20	1.10E−02						7	1.42	8.93E−04				
ola04672	Intestinal immune network for IgA production	7	1.60	7.74E−05						5	0.75	2.23E−02		8	1.62	2.37E−05				
ola00380	Tryptophan metabolism	4	0.92	7.84E−02		6	1.44	2.23E−03										12	0.52	0.021
ola00830	Retinol metabolism	5	1.14	1.87E−02										6	1.21	7.74E−03				
ola04630	Jak–STAT signaling pathway	9	2.06	5.01E−03		11	2.64	1.65E−04												
ola04130	SNARE interactions in vesicular transport																	12	0.52	0.006
ola00400	Phenylalanine, tyrosine, and tryptophan biosyntheses					3	0.72	1.46E−02												
ola01100	Metabolic pathways																	208	8.93	0.000
ola00100	Steroid biosynthesis																	8	0.34	0.019
ola00120	Primary bile acid biosynthesis																	7	0.30	0.034
ola00240	Pyrimidine metabolism																	26	1.12	0.001
ola00360	Phenylalanine metabolism					3	0.72	4.97E−02												
ola00480	Glutathione metabolism																	14	0.60	0.044
ola00563	Glycosylphosphatidylinositol(GPI)-anchor biosynthesis																	7	0.30	0.027
ola04920	Adipocytokine signaling pathway																	19	0.82	0.038

### 3.4 Interactions between nAChR and DINO and its metabolites

**Fig. 3:**
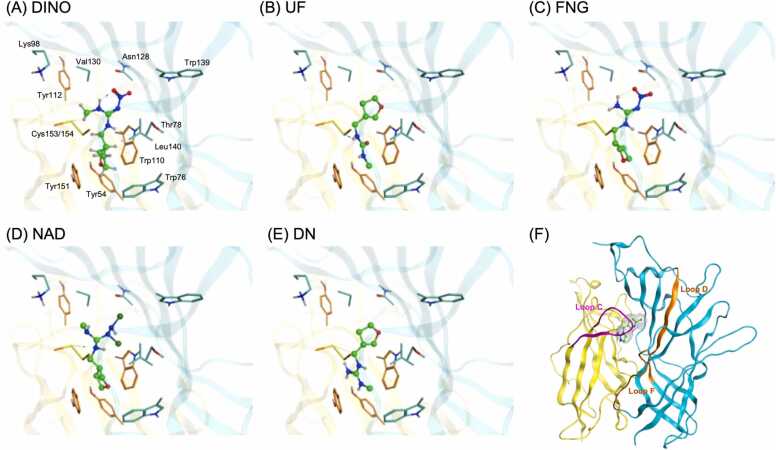
**Binding modes of DINO and its metabolites to the medaka α4β2 nicotinic acetylcholine receptor (nAChR).** DINO and key amino acid residues interacting with each ligand are shown (A-E). Hydrogen bonds and CH–π interactions are indicated by dotted lines. Ligands are shown as balls and sticks and colored by atom type: Carbon atoms in green, oxygen in red, and nitrogen in blue. Relevant amino acid residues are colored by atom type: Carbon atoms are indicated as orange sticks for α4 and cyan sticks for β2. α4 is indicated in yellow, and β2 is indicated in cyan (F).

To investigate the interactions between medaka α4β2 nAChR and DINO, we constructed a homology model of nAChR using the crystal structure of the insect ACh-binding protein in complex with desnitro-imidacloprid as a template (Fig. 3F). The cavity volume of the ligand-binding site was 142 Å^3^. Docking simulation analysis revealed that the dock-induced S-scores of DINO, UF, FNG, NAD, and DN were –6.854, –6.004, –6.602, –6.860, and –6.644 kcal/mol, respectively (Table S2). Binding modes of DINO and its metabolites to the medaka α4β2 nAChR are shown in Fig. 3A−3E and Fig. S3. The key amino acid residues participating in the hydrogen bond interactions of DINO were the Val130 and Leu140 backbones and the Cys154 side chain (Fig. 3). NAD, which had the lowest S-score, interacted with the Trp110 backbone and Cys154 side chain via hydrogen bonding. Hydrogen bonds were formed with Trp110 and Cys153 in UF and Val130 and Leu140 in FNG. DN formed only a hydrogen bond with Cys154. For DINO and FNG binding, Trp110 and Tyr158 contributed to ligand preference by facilitating CH–π interactions.

## 4 Discussion

DINO exhibits high water solubility and hydrolytic stability, leading to its photolytic degradation in water [46], [47]. Its metabolites exhibit potentially toxicity. However, studies on the toxicological effects of DINO in fish are limited. Therefore, in this study, we investigated the effects of DINO and its metabolites on the embryonic development and differentially expressed genes in medaka and evaluated their interactions with medaka α4β2 nAChR.

In this study, we used the nsPEF technique to incorporate DINO and its metabolites into medaka embryos. Firstly, we confirmed that the nsPEF technique reliably incorporates DINO into the embryos (Fig. S2). As the metabolites were synthetic compounds, it was not possible to obtain a sufficient quantities of the reagents required to measure their concentrations in the eggs. The phenotypic analysis results for DINO are shown in Fig. 2 and Table 1. No significant differences were observed in the incidence of thrombosis and mean hatching date. Post-hatchling malformations were observed in all the treatment groups. The incidence of malformed larvae was approximately 10% in all treatment groups, with significant differences between the control and NAD treatment groups. No significant differences were observed between the DINO and metabolite treatment groups. Chemical-specific malformations were not observed in any group. These results suggest that DINO and its metabolites exert only weak effects on the early development of medaka but cause malformations, and that their toxicity is comparable to that of the parent compound. Furthermore, nsPEF technique enabled the evaluation of the biological impact of metabolites. In a neurobehavioral toxicity study in zebrafish larvae, exposure to 10 and 100 µM DINO was hypoactive, relative to that observed in the controls, with only the 100 µM group showing impaired survival to adulthood [48]. Wagner et al. [49] reported that fipronil, a phenylpyrazole pesticide, causes deformities in medaka eggs [49]. Therefore, the early developmental stages of fish are vulnerable to neonicotinoids, and DINO and its metabolites adversely affect fish larvae.

The stereoselective behavior of DINO in a water-sediment microcosmic system indicates that the main metabolites of DINO enantiomers in zebrafish are UF and DN [28]. UF and DN exhibit similar toxicities and induce the overproduction of reactive oxygen species, resulting in oxidative stress in earthworms [27]. In the present study, DINO metabolites induced malformations in medaka larvae comparable to those induced by DINO. All DINO metabolites used in this study contained a (tetrahydro−3-furyl)-methyl group. Therefore, toxicity of DINO and its metabolites is possibly related to the (tetrahydro−3-furyl)-methyl group, with this chemical group, including its metabolites, adversely affecting medaka development.

In the present study, RNA-seq was performed to investigate the effects of DINO and its metabolites on medaka development. DINO competitively binds to the nAChR, thereby preventing acetylcholine from binding to its receptor. Functional pathway analysis showed that genes altered by DINO and its metabolites were commonly involved in neuroactive ligand–receptor interactions. The neuroactive ligand–receptor interaction signaling pathway includes neurotransmitter receptors, which play crucial roles in neuronal function. This pathway is often associated with developmental toxicity in fish [33], [50], [51]. Recent metabolomic analyses of honeybee larvae have indicated that disturbances in the neuroactive ligand–receptor pathway and energy metabolism are key mechanisms of DINO toxicity [52]. Expression profiles of circular RNAs in the brains of honeybees exposed to DINO indicate enrichment of the neuroactive ligand–receptor pathway [53]. These findings suggest that exposure to DINO and its metabolites disrupts neurotransmitter homeostasis in medaka larvae. Cytokine-cytokine receptor interactions were also affected by DINO and its metabolites, except in the DN-exposed medaka embryos. Cytokine–cytokine receptor interactions regulate the normal development and function of multiple cell types, particularly blood and immune cells, and their perturbations are directly associated with many diseases [54]. Studies of the stereoselectivity of DINO toxicity in zebrafish revealed, in agreement with our results, that this pathway was remarkably enriched in both the transcriptomic and proteomic profiles of R- and S-dinotefuran [28]. These molecular pathways play important roles in medaka–insecticide interactions, resulting in malformations. In this study, metabolic pathway, steroid biosynthesis, and GPI-anchor biosynthesis pathways were enriched only in the DN treatment group. Although its mechanism of action differs from that of the other metabolites, DN exhibits developmental toxicity in medaka embryos.

To predict the binding affinity energy of DINO and its metabolites to nAChR, we modeled the 3D structure of the medaka α4β2 nAChR. This subunit structure is conserved in both the aromatic and vicinal cysteine residues. The calculated volume of the medaka nAChR ligand-binding cavity was 141.9 Å^3^. Ligands were mainly confined to loop C in the α4 subunit and loops D and F in the β2 subunit (Fig. 3F). This binding pocket is the agonist-binding site for nicotine in the crystal structure of human nAChR [55]. Neonicotinoids and some nAChR-targeting alkaloids, such as cotinine, anatabine, and methylanatabine, have been docked at this binding site [56], [57]. Our molecular docking models closely overlapped with previously reported receptor–ligand complexes. The order of the interaction potentials of DINO with medaka α4β2 nAChR was as follows: NAD > DINO > DN > FNG > UF. Notably, NAD, an amino substituent, showed a higher binding affinity than DINO. The nicotinic receptor recognizes nicotinoids as agonists via cation–π interactions [6]. Here, NAD docking position did not exhibit cation–π interactions. However, hydrogen bonds were formed between Trp110 and Cys154. Except for the highest interaction energy in the UF, the predicted affinities exhibited a trend similar to that of the toxic potencies of DINO during embryonic development. Although UF showed a high interaction energy with DINO, hydrogen bonds were formed with Trp110 and Cys153, similar to NAD, suggesting that UF also interacts with nAchR. These results suggest that compounds with high affinity for α4β2 nAChR impact the embryonic development in medaka.

## 5 Conclusions

In conclusion, no acute toxicity was detected during the early developmental stages of medaka exposed to DINO and its metabolites; however, some larvae malformed after hatching. Transcriptome sequencing revealed that neuroactive ligand–receptor interactions play an important role in DINO-induced developmental toxicity. Additionally, *in silico* analysis revealed that DINO metabolites could bind to medaka nAchR. The teratogenicity of these metabolites was almost identical to that of DINO, suggesting that the toxicities of the four metabolites used in this study did not change due to metabolism. This study provides valuable data on the adverse effects of DINO metabolites on the developmental toxicity responses of fish to neonicotinoid pesticides. Future studies are needed to clarify the toxicity risks associated with DINO metabolites in other aquatic organisms.

## CRediT authorship contribution statement

**Tadashi Okobira:** Writing – review & editing, Methodology, Data curation. **Keisuke Kato:** Writing – review & editing, Resources. **Koji Arizono:** Writing – review & editing, Project administration, Funding acquisition, Conceptualization. **Hiroshi Ishibashi:** Writing – review & editing, Data curation. **Daishi Inoue:** Writing – review & editing, Methodology, Formal analysis, Data curation, Conceptualization. **Masashi Hirano:** Writing – original draft, Methodology, Formal analysis, Data curation, Conceptualization. **Keisuke Takahashi:** Writing – review & editing, Resources. **Masaya Uchida:** Writing – review & editing, Methodology, Formal analysis, Data curation, Conceptualization. **Nobuaki Tominaga:** Writing – review & editing, Project administration, Funding acquisition, Conceptualization.

## Declaration of Competing Interest

The authors declare that they have no known competing financial interests or personal relationships that could have appeared to influence the work reported in this paper.

## Data Availability

Data will be made available on request.
