# Supplementary material for: Effects of dinotefuran and its metabolites on the early life stage development of medaka (Oryzias latipes): In vivo and in silico studies
Source: Toxicol Rep. 2026 Jun 12;17:102293. doi: 10.1016/j.toxrep.2026.102293 (PMC13284504; doi:10.1016/j.toxrep.2026.102293)
Supplement: Supplementary file 1 — Supplementary material [file mmc1.docx]

**Supplementary data**

**Effects of dinotefuran and its metabolites on the early life stage development of medaka (*Oryzias latipes*): *In vivo* and *in silico* studies**

Masashi Hirano^1^, Daishi Inoue^2^, Masaya Uchida^2^, Keisuke Takahashi^3^, Keisuke Kato^3^, Tadashi Okobira^2^, Hiroshi Ishibashi^4^, Koji Arizono^5^, and Nobuaki Tominaga^2,*^

**AUTHOR ADDRESS:**

^1^ Department of Food and Life Sciences, School of Agriculture, Tokai University, Mashiki-Machi, Kamimashiki-Gun, Kumamoto 861-2205, Japan

^2^ Department of Creative Engineering, National Institute of Technology, Ariake College, 150 Higashi-Hagio, Omuta, Fukuoka 836-8585, Japan

^3^ Faculty of Pharmaceutical Sciences, Toho University, 2–2-1 Miyama, Funabashi, Chiba 274–8510, Japan

^4^ Graduate School of Agriculture, Ehime University, 3-5-7 Tarumi, Matsuyama, Ehime 790-8566, Japan

^5^ Graduate School of Pharmaceutical Sciences, Kumamoto University, 5-1 Oe, Chuo-ku, Kumamoto 862-0973, Japan

*Corresponding author: Nobuaki Tominaga, Department of Creative Engineering, National Institute of Technology, Ariake College, 150 Higashi-Hagio, Omuta, Fukuoka 836-8585, Japan.

E-mail: tominaga@ariake-nct.ac.jp, Tel & FAX: +81-944-53-8747


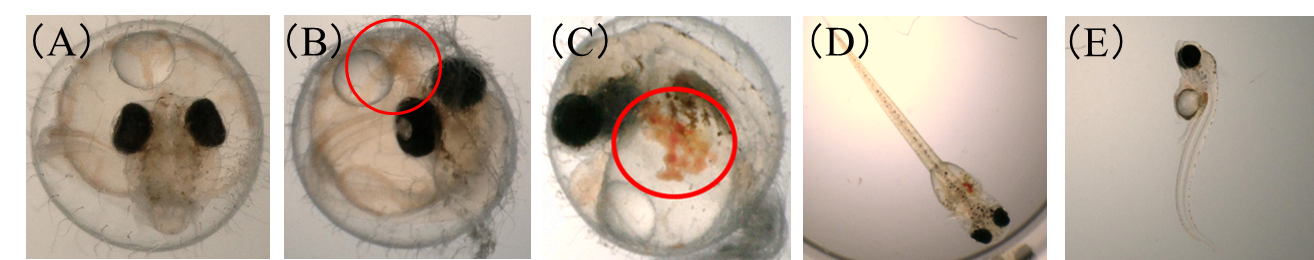


**Supplementary Fig. S1. Representative optical images of the deformed medaka embryos and larvae.** (A) Normal. (B) Hypercardia. (C) Thrombus. (D) Normal. (E) Deformation.


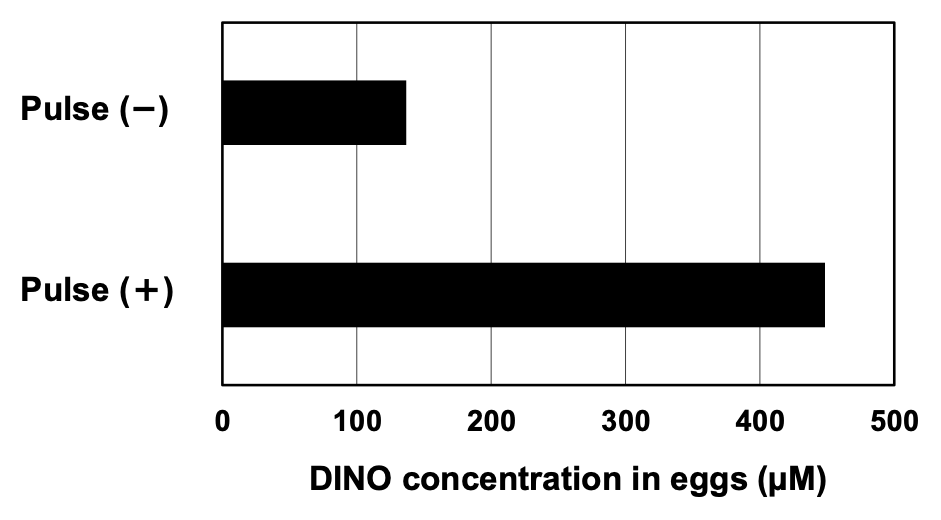


**Supplementary Fig. S2. DINO concentrations in eggs exposed to 2 mM DINO for 2 h, with or without nsPEF treatment.**


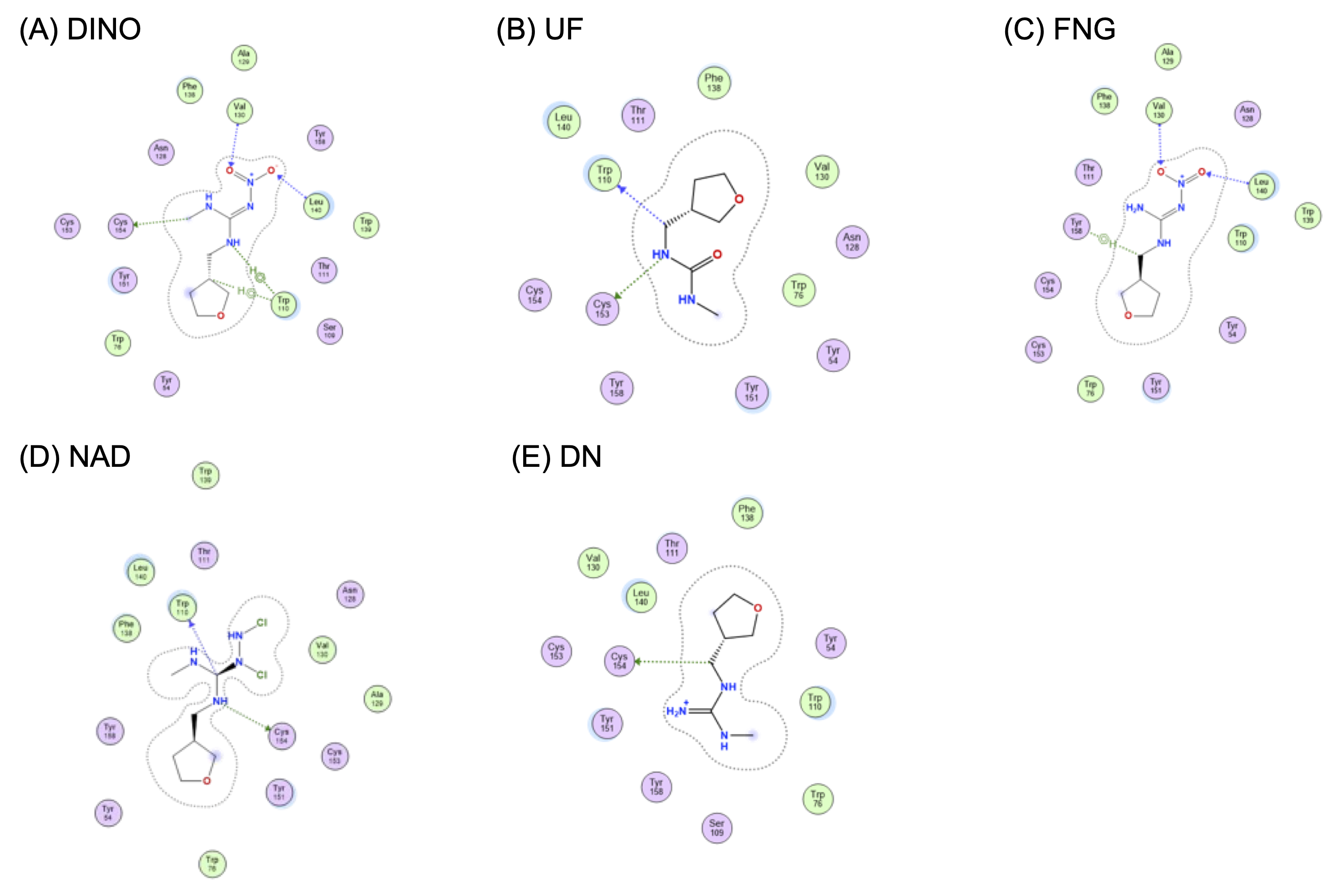


**Supplementary Fig. S2. Two-dimensional (2D) visualization of dinotefuran (DINO) interactions with the binding site of medaka α4β2 nicotinic acetylcholine receptor (nAChR). Representative optical images of the deformed medaka embryos and larvae.** Interactions between DINO and receptor residues are indicated by dashed lines.

**Supplementary Table S1. Analytical conditions for ultra-performance liquid chromatography-mass spectrometry (UPLC-MS) for the identification of dinotefuran (DINO) in medaka embryos.**

| Column | BEH C18 (1.7 µm, 2.1 × 50 mm) |
| --- | --- |
| Mobile phase | A: 0.1 % Formic acid in water B: 0.1 % Formic acid in acetonitrile A:B = 95:5 (0 min) → 95:5 (1 min)  → 5:95 (13 min) → 75:25 (13.1 min)  → 75:25 (14 min) → 75:25 (40 min)  → 95:5 (40.1 min) |
| Flow rate | 0.3 mL/min |
| Column temp. | 40 ℃ |
| Injection vol. | 10 µL |

**Supplementary Table S2. *In silico* docking simulation analysis and parameters of medaka α4β2 nicotinic acetylcholine receptor (nAChR) interactions with DINO.**

| Compound | S-score (kcal/mol) | Hydrogen bonding | | CH–π |
| --- | --- | --- | --- | --- |
|  |  | Amino acids | Interactions with the backbone (B) or residue side chain (S) |  |
| DINO | –6.854 | Val130 (β2) | B | Trp110 |
|  |  | Leu140 (β2) | B |  |
|  |  | Cys154 (α4) | S |  |
| UF | –6.004 | Trp110 (α4) | B |  |
|  |  | Cys153 (α4) | S |  |
| FNG | –6.602 | Val130 (β2) | B | Tyr158 |
|  |  | Leu140 (β2) | B |  |
| NAD | –6.860 | Trp110 (α4) | B |  |
|  |  | Cys154 (α4) | S |  |
| DN | –6.644 | Cys154 (α4) | S |  |
